# Molecular Epidemiology and Transmission Dynamics of the HIV-1 Epidemic in Ethiopia: Epidemic Decline Coincided With Behavioral Interventions Before ART Scale-Up

**DOI:** 10.3389/fmicb.2022.821006

**Published:** 2022-02-25

**Authors:** Dawit Assefa Arimide, Luis Roger Esquivel-Gómez, Yenew Kebede, Sviataslau Sasinovich, Taye Balcha, Per Björkman, Denise Kühnert, Patrik Medstrand

**Affiliations:** ^1^Department of Translational Medicine, Lund University, Malmo, Sweden; ^2^TB/HIV Department, Ethiopian Public Health Institute, Addis Ababa, Ethiopia; ^3^Transmission, Infection, Diversification and Evolution Group, Max-Planck Institute for the Science of Human History, Jena, Germany; ^4^Africa Centre for Disease Prevention and Control, Africa Union Commission, Addis Ababa, Ethiopia

**Keywords:** effective reproductive number, effective population size, birth–death model, phylodynamic, HIV-1 epidemic, transmission cluster, behavioral intervention, Ethiopia

## Abstract

**Background:**

Ethiopia is one of the sub-Saharan countries hit hard by the HIV epidemic. Previous studies have shown that subtype C dominates the Ethiopian HIV-1 epidemic, but the evolutionary and temporal dynamics of HIV-1 in Ethiopia have not been closely scrutinized. Understanding the evolutionary and epidemiological pattern of HIV is vital to monitor the spread, evaluate and implement HIV prevention strategies.

**Methods:**

We analyzed 1,276 Ethiopian HIV-1 subtype C polymerase (*pol* sequences), including 144 newly generated sequences, collected from different parts of the country from 1986 to 2017. We employed state-of-art maximum likelihood and Bayesian phylodynamic analyses to comprehensively describe the evolutionary dynamics of the HIV-1 epidemic in Ethiopia. We used Bayesian phylodynamic models to estimate the dynamics of the effective population size (N_e_) and reproductive numbers (R_e_) through time for the HIV epidemic in Ethiopia.

**Results:**

Our analysis revealed that the Ethiopian HIV-1 epidemic originated from two independent introductions at the beginning of the 1970s and 1980s from eastern and southern African countries, respectively, followed by epidemic growth reaching its maximum in the early 1990s. We identified three large clusters with a majority of Ethiopian sequences. Phylodynamic analyses revealed that all three clusters were characterized by high transmission rates during the early epidemic, followed by a decline in HIV-1 transmissions after 1990. R_e_ was high (4–6) during the earlier time of the epidemic but dropped significantly and remained low (R_e_ < 1) after the mid-1990. Similarly, with an expected shift in time, the effective population size (N_e_) steadily increased until the beginning of 2000, followed by a decline and stabilization until recent years. The phylodynamic analyses corroborated the modeled UNAIDS incidence and prevalence estimates.

**Conclusion:**

The rapid decline in the HIV epidemic took place a decade before introducing antiretroviral therapy in Ethiopia and coincided with early behavioral, preventive, and awareness interventions implemented in the country. Our findings highlight the importance of behavioral interventions and antiretroviral therapy scale-up to halt and maintain HIV transmissions at low levels (R_e_ < 1). The phylodynamic analyses provide epidemiological insights not directly available using standard surveillance and may inform the adjustment of public health strategies in HIV prevention in Ethiopia.

## Introduction

The human immunodeficiency virus type 1 (HIV-1) is one of the most devastating infectious diseases in human history (UNAIDS, 2020). At the end of 2020, an estimated 38 million people were living with HIV/AIDS worldwide. Sub-Saharan Africa, the region where HIV-1 emerged during the 1920s, remains the most affected region, accounting for close to 70% of people living with HIV worldwide (Faria et al., 2014; UNAIDS, 2020). Despite the large-scale roll-out of antiretroviral treatment (ART), HIV incidence remains high, mainly in sub-Saharan Africa (UNAIDS, 2020). Ethiopia is one of the many sub-Saharan countries that was severely affected by the HIV epidemic.

HIV-1 is classified into four phylogenetically distinct groups: M (main), N (non-M, non-O), O (outlier), and P (pending), each representing different zoonotic cross-species transmissions of simian immunodeficiency viruses from non-human primates to humans (Sharp and Hahn, 2011; Faria et al., 2014; Giovanetti et al., 2020). Group M is the most prevalent, accounts for more than 95% of all the HIV-1 infections and is divided into 10 subtypes (A–D, F–H, and J–L), more than 102 different circulating recombinant forms (CRFs), and numerous unique recombinant forms (URFs; Hemelaar et al., 2019; Giovanetti et al., 2020). Subtype C is currently the dominant HIV-1 subtype and is responsible for nearly half all HIV-1 infections globally (Hemelaar et al., 2019). Although found worldwide, no official assignment of subtype C strains into phylogenetic sub-subtypes has been made. However, several distinct genetic clades associated with geography have been defined, the southern African clades (C-SA) and the eastern African clade (C-EA). Strains of the C-EA clade and a sub-clade of C-SA, termed C′-ET, are most prevalent in Ethiopia (Thomson and Fernandez-Garcia, 2011; Arimide et al., 2018).

The first HIV-1 infection and AIDS case report in Ethiopia was in 1984 and 1986, respectively (Lester et al., 1988; Tsega et al., 1988). Initially, the epidemic was concentrated to urban areas and along major commercial routes. Serology surveys revealed high prevalence (17%–55%) among risk populations (e.g., female sex workers: FSWs, long-distance truck drivers: LDTD, and soldiers; Mehret, 1990; Mebret et al., 1990). However, after introduction of antiretroviral therapy (ART) in public health care in 2005, the prevalence among the general population decreased and stabilized at significantly lower levels while the prevalence remained high in risk populations (EPHI, 2014).

The HIV epidemic in Ethiopia is considered a generalized epidemic with heterosexual transmission being the dominant mode of transmission (Kebede et al., 2000). Since 1985, Ethiopia has implemented several community-based HIV prevention programs to improve knowledge about the infection and mode of transmission, and interventions to reduce engagement in risk behavior (Mebret et al., 1990; Okubagzhi and Singh, 2002). However, the epidemiological dynamics and their correlations with introduction of various HIV prevention and interventions programs have not been characterized.

Similar to many low-income countries, epidemiological data regarding HIV from Ethiopia are sparse and incomplete, making surveillance of the HIV epidemic challenging. The increased availability of HIV genetic sequencing data and the development of phylogenetic and phylodynamic tools has enabled the use of molecular epidemiology analysis to describe the transmission dynamics and evolutionary history of HIV (Yusim et al., 2001; Delatorre and Bello, 2012; Mir et al., 2018; Vasylyeva et al., 2019). Previous studies in Ethiopia have shown that subtype C dominates the Ethiopian HIV epidemic and have provided valuable insight into HIV genetic diversity, its origins, and epidemic dynamics, but are limited in study participant numbers and geographic and temporal representation (Abebe et al., 2001a,b; Pollakis et al., 2003; Tully and Wood, 2010; Delatorre and Bello, 2012; Mir et al., 2018). Here, we used HIV-1 subtype C *pol* gene sequences collected from different regions of Ethiopia between 1986 and 2017. We employed state-of-the-art phylogenetic and phylodynamic methods, including both Bayesian coalescent and birth–death modeling, to elucidate evolutionary trajectories and temporal dynamics of the HIV-1 epidemic in Ethiopia.

## Materials and Methods

### Baseline HIV-1 Drug Resistance Survey

We conducted a prospective HIVDR survey among antiretroviral-naïve adults in St. Paul General Specialized Hospital located in Addis Ababa, Ethiopia, in 2011. We performed the study according to the WHO-recommended survey methodology (Jordan et al., 2008). Treatment-naïve adults (>18 years) eligible to start ART at the St. Paul Generalized Specialized Hospital were consecutively enrolled. Whole blood specimens were collected and transported to the Ethiopia Public Health Institute (EPHI), the national HIV laboratory, and WHO-accredited laboratory for viral load testing and HIVDR genotyping.

HIV genotyping was done using an in-house assay as described previously (Arimide et al., 2018). Briefly, a 1,084 base-pair fragment of HIV-1 *pol* (corresponding to positions 2,243–3,326 of HXB2; GenBank Accession Number: K03455) comprising amino acids 6–99 of the protease and 1–251 of the reverse transcriptase was obtained by RT-PCR and nested PCR. The purified PCR fragments were then sequenced and analyzed on the ABI 3500xl Genetic Analyzer (Applied Biosystems, Foster City, CA, United States). Sequence assembly and editing were performed using the RECall V 2.0 HIV-1 sequencing analysis tool (University of British Columbia, Vancouver, Canada; Woods et al., 2012). All sequences reported in this study have been deposited in GenBank under Accession Numbers OL598713-OL598856.

### Study Population and Sequence Dataset

We used the dataset of newly sequenced HIV-1 *pol* sequences and retrieved all publicly available Ethiopian HIV-1 subtype C *pol* sequences (matching pos. 2,243–3,326 relative of HXB2) from the Los Alamos National Laboratory (LANL) HIV Sequence database^1^ (Date of access, December 2019). The quality of HIV-1 sequences was verified using the online Quality Control program of the LANL HIV sequence database (see Footnote 1) and sequences with stop codons, frameshifts, and poor quality were removed. We retained only one sequence per patient and selected the earliest sequence for patients with multiple sequences.

We removed duplicate sequences and sequences with potential contamination using the ElimDupes online tool from LANL. Moreover, to identify Ethiopian country-specific transmission clusters, we included a dataset of similar sequences from GenBank by identifying the 10 genetically closest GenBank sequences with BLAST for each Ethiopian HIV-1 subtype C sequence (Altschul et al., 1990; Mount, 2007). We only included sequences of 950 nucleotides or longer with known isolation dates and country of isolation in the analysis, since this 950-bp region has sufficient signal to reconstruct transmission links among infected individuals (Hué et al., 2004).

### HIV-1 Subtyping

Initial explorative HIV-1 subtyping was performed using the online automated subtyping tools REGA v3.0 (Pineda-Peña et al., 2013), COMET v2.2 (Struck et al., 2014), and RIP (Martin et al., 2010). Putative intra-subtype recombinant sequences were detected using jpHMM (jumping profile Hidden Markov Model)^2^ (Schultz et al., 2009; Arimide et al., 2018). Only non-recombinant sequences were used for the analysis. Final subtyping was determined by maximum likelihood (ML) phylogenetic tree analysis with subtype reference sequences (Arimide et al., 2018).

### Maximum Likelihood Phylogenetic Analyses

A multiple sequence alignment was obtained using MAFFT V. 7 (Katoh and Standley, 2013) and was then manually edited using BioEdit V7.0.9.0 (Hall, 1999) until a non-redundant codon alignment was obtained. To avoid the effect of drug-induced convergent evolution, positions of identified mutations causing or contributing to HIVDR were removed from the alignment, resulting in a final alignment of 909 bp (Wensing et al., 2016).

The initial ML phylogenetic tree was constructed using an online version of PhyML (Guindon et al., 2010) under the GTR + I + Γ4 (general time-reversible nucleotide substitution model using the estimated proportion of invariable sites and four gamma categories). Heuristic tree search was performed using the SPR branch-swapping algorithm. Branch support was determined with aLRT-SH (approximate likelihood ratio test Shimodaira–Hasegawa-like) implemented in PhyML (Guindon et al., 2010). A branch in the phylogeny with an aLRT-SH value ≥0.9 was considered significant (Guindon et al., 2010; Esbjörnsson et al., 2016). The ML trees were visualized using FigTree v1.4.3 (Rambaut, 2016).

Our initial ML phylogenetic trees were constructed using the combined dataset of all Ethiopian sequences and sequences from the BLAST search. To comprehensively describe the HIV-1 subtype C circulating in Ethiopia, the dataset was divided into two based on phylogenetic branch support, the C-EA and C-ET clades.

### Analysis of Transmission Clusters

Separate transmission cluster analysis was performed for the two data sets using the ML phylogenetic analysis implemented by IQ-TREE under GTR + I + Γ4 as selected as the best fitting substitution model for the dataset using jModelTest v2.1.7 and with 1,000 replicates for the aLRT-SH test (Nguyen et al., 2015). Clusters with an aLRT-SH support ≥0.9 were considered significant (Guindon et al., 2010; Esbjörnsson et al., 2016). A transmission cluster was defined as a cluster in the ML phylogeny from root to tips (Esbjörnsson et al., 2016; Hassan et al., 2017; Sallam et al., 2017; Arimide et al., 2018). Clusters with an aLRT-SH-support of ≥0.9 that had a majority (at least 80%) of Ethiopia sequences were considered an Ethiopian transmission cluster. Transmission clusters were also defined based on their sizes (number of sequences/cluster), into dyads (two sequences), medium-sized clusters/networks (3–14 sequences), and large clusters (≥15 sequences; Aldous et al., 2012; Esbjörnsson et al., 2016).

To determine whether there was phylogenetic clustering by geographic region, viral sequences were grouped into six geographic regions (sequence collection location). The strength of association between the geographic location and the phylogeny was determined using two phylogeny–trait association statistics, the parsimony score (PS) and the association index (AI) tests, both of which were implemented in the Bayesian Tip-association Significance testing (BaTS) program (Parker et al., 2008). A significance level of *p* < 0.05 was used in both statistics.

### Estimating Temporal Signal

For each cluster, we assessed the temporal signal of the data sets by performing root-to-tip genetic distance using TempEst (Rambaut et al., 2016). Clusters that had a positive correlation between genetic diversity and time were considered for further analysis.

### Estimating Viral Phylodynamic History

The birth–death skyline model (BDSKY; Stadler et al., 2012, 2013) implemented in BEAST2 v 2.6.2 was used to quantify epidemic growth through time described by changes in the effective reproductive number (R_e_) which is the average number of secondary infections from an infected individual at any given time during the epidemic (Bouckaert et al., 2019; Vasylyeva et al., 2019). We used a lognormal distribution prior, LogNorm (0,1), for the effective reproductive number with the upper bound of 10, and a LogNorm (0,1) prior for the become uninfectious rate (*δ*) in units per year (i.e., the inverse of the time duration of being infectious in a unit of years). We used *δ* = 0.2, corresponding to a 5-year duration of the infectious period, as the mean of the distribution. In order to account for the uneven number of sequences per year, we employed a different sampling probability (*rho*) prior for each year, using a *beta* distribution with a mean equal to the number of samples divided by the reported number of HIV cases in the country for that year. We estimated the change in R_e_ for six equally spaced intervals between the time to most recent common ancestor (tMRCA) and the most recent sampling year.

Phylodynamic analyses were also performed using the Bayesian Skygrid coalescent tree prior, implemented in BEAST 1.10,4 (Gill et al., 2013; Suchard et al., 2018; Hill and Baele, 2019), to estimate changes in effective population size (N_e_) through time and estimate the population growth rates (*r*, years^−1^) by using a logistic growth coalescent tree prior. Analyses were performed using the GTR + I + Γ4 nucleotide substitution model. The temporal scale of the evolutionary process was estimated using a relaxed uncorrelated molecular clock model with an underlying lognormal distribution with normal priors. This allowed the estimation of the evolutionary rate (*μ*, nucleotide substitutions per site per year, s/s/y), the age of the most recent common ancestor (tMRCA, years), and the phylodynamic parameters.

For each of the two phylodynamic approaches, we ran three independent Markov Chain Monte Carlo (MCMC) chains until all associated parameters converged to ensure good mixing (ESS > 200) after discarding the first 10% of the MCMC chains. The convergence of the MCMC was inspected visually and by calculating the ESS for each parameter using Tracer v 1.7.5 (Rambaut et al., 2018). We used LogCombiner to combine the different independent results (log and corresponding tree file) from the multiple chains (Drummond et al., 2012). We used the bdskytools package^3^ in R to plot the results of the BDSKY analysis.

### Ethical Approval

We obtained ethical approval from the Research and Ethical Clearance Committee of EPHI and the National Health Research Ethics Review Committee of the Ministry of Science and Technology of Ethiopia. All participants for the baseline HIV drug resistance survey provided written informed consent to participate in the study.

## Results

### Study Population and Initial Phylogenetic Analysis

**Figure 1:**
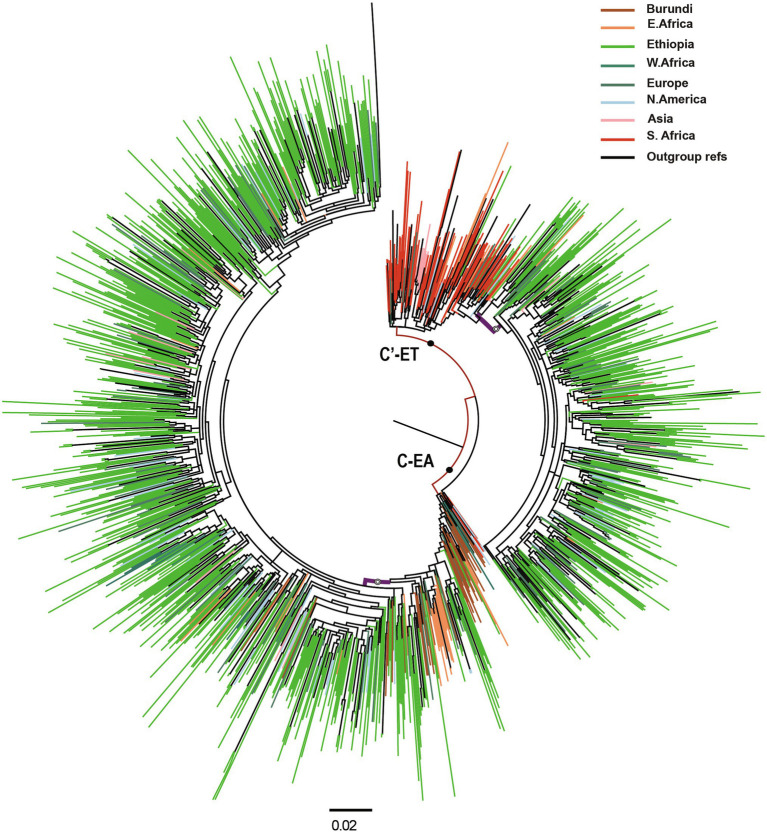
Maximum likelihood (ML) phylogenetic tree of HIV-1 subtype C pol sequences (*n* = 1,333). Maximum likelihood phylogenetic tree constructed using 877 Ethiopian subtype C *pol* sequences collected 1986–2017 and 456 global subtype C *pol* sequences. Colored tips are according to the geographic origin of sequences, as indicated in the legend in the top right corner. Branches defining the major clades (C-EA and C′-ET) are those indicated with a filled circle, having branch support (aLRT-SH) >0.9. The Ethiopian clusters are indicated by open circles, having branch support (aLRT-SH) >0.9. The scale bar represents 0.02 substitutions/site.

We retrieved 1,132 Ethiopian *pol* sequences from LANL, collected from different parts of the country from 1986 to 2017. Additionally, we included 144 HIV-1 *pol* sequences from the baseline HIVDR survey. The combined dataset (*n* = 1,276 sequences) contained 399 putative recombinant sequences, which were removed from further analysis. We further included a dataset of similar sequences from GenBank by identifying the 10 genetically closest GenBank sequences with BLAST for each of the 877 non-recombinant Ethiopian HIV-1 subtype C sequences in the study. The final combined dataset contained 1,333 non-recombinant HIV-1 subtype C *pol* sequences (877 Ethiopian and 456 global), which were used for phylogenetic analysis. The ML phylogenetic tree identified two distinct and well-supported clades, the C-EA and C′-ET clades (Figure 1). Among the 877 Ethiopian sequences included in the analysis, the C-EA clade represented 567 (65.0%) of the sequences, while 310 (35.0%) belonged to the C′-ET clade.

Most of the Ethiopian C-EA sequences were found in one large cluster (aLRT = 0.87), and only 33 Ethiopian C-EA sequences fell outside this cluster. Sequences of the global dataset intermixed with the Ethiopian sequences and represented sequences obtained most frequently (*N* = 67, 33.3%) in other East African countries, North America, and Europe. Sequences from Burundi dominated the basally located sequences. In the case of the second major clade, the majority of the Ethiopian C′-ET sequences (95.5%) formed a well-supported sub-clade (aLRT = 0.92), branching off from the basally located sequences. Southern African countries’ sequences were intermixed (*N* = 121, 47.1%) with the Ethiopian C′-ET sequences, but they were most prominent at the base of the clade.

### Transmission Cluster Analysis

**Figure 2:**
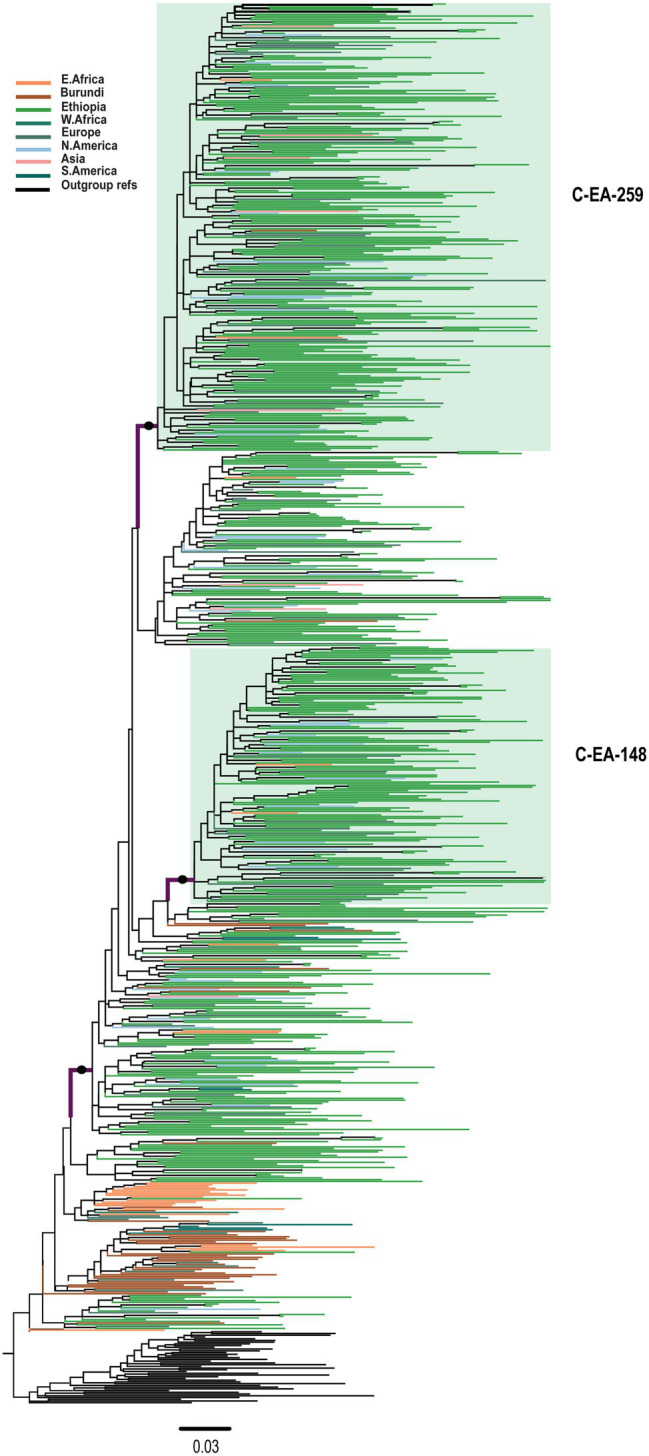
Maximum likelihood phylogenetic tree of HIV-1 C-EA clade *pol* sequences (*n* = 810). Maximum likelihood phylogenetic tree constructed using 567 Ethiopian subtype C *pol* sequences collected between 1986 and 2017, 201 global subtype C *pol* sequences, and 42 reference sequences. Colored tips are according to the geographic origin of sequences, as indicated in the legend in the top left corner. The C-EA-259 and C-EA-148 clusters are highlighted in green, corresponding to a branch support aLRT-SH >0.9 and >80% Ethiopian sequence. The filled circle defining the C′-ET clade represents an aLRT-SH >0.9. The tree was rooted using the C′-ET reference sequences. The scale bar represents 0.03 nucleotide substitutions per site.

We inferred transmission clusters by separate ML phylogenetic tree analyses of the two clades (C-EA and C′-ET). For C-EA, the ML phylogenetic tree contained a total of 810 sequences (with reference sequences) and identified two large well-supported clusters of 259 (C-EA-259) and 148 sequences (C-EA-148). The C-EA-259 cluster (aLRT = 0.93) contained 213 Ethiopian (82.2% of the sequences of the cluster) and 46 non-Ethiopian sequences, collected 1988–2017, and the C-EA-148 cluster (aLRT = 0.95) contained 124 Ethiopian (83.8%) sequences and 24 non-Ethiopian sequences, collected 1996–2017 (Figure 2). Moreover, we identified six networks (medium-sized clusters) and nine dyads.

**Figure 3:**
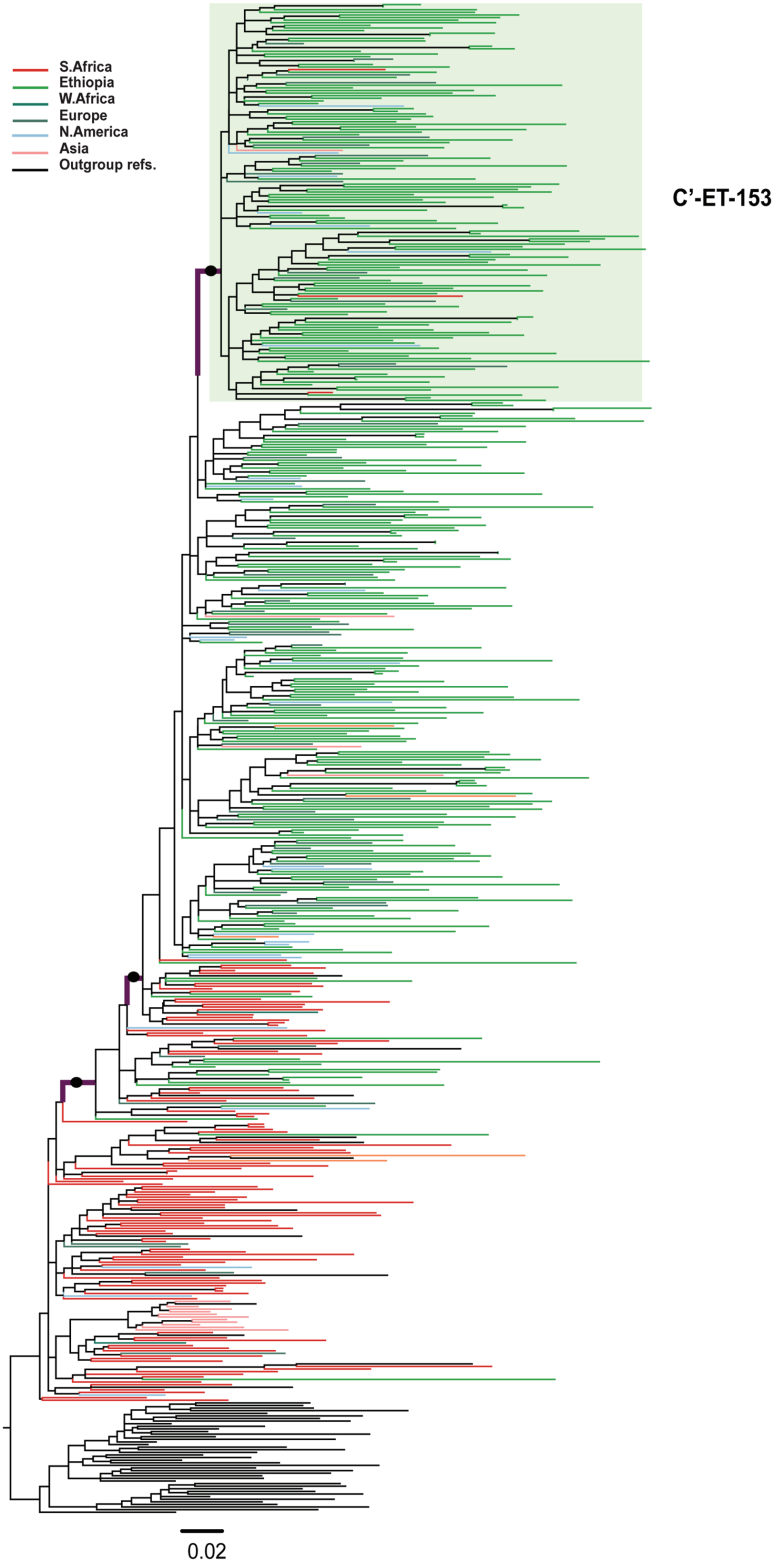
Maximum likelihood phylogenetic tree of HIV-1 C′-ET clade *pol* sequences (*n* = 580). Maximum likelihood phylogenetic tree constructed using 310 Ethiopian subtype C *pol* sequences collected 1986–2017, 227 global subtype C *pol* sequences, and 43 reference sequences. Colored tips are according to the geographic origin of sequences, as indicated in the legend in the top left corner. The C′-ET-153 cluster, highlighted in a green shade, is defined by branch support (aLRT-SH) of >0.9 and >80% Ethiopian sequences. The branches with filled circles possibly defining the C′-ET clade have branch support (aLRT-SH) >0.9. The tree was rooted using the C-EA reference sequences. The scale bar represents 0.02 nucleotide substitutions per site.

Among the 580 C′-ET sequences, we identified one well-supported (aLRT = 0.96) large cluster containing 153 sequences [C′-ET-153; 124 Ethiopian (81.0% of the sequences in the cluster)], and 29 non-Ethiopian sequences collected 1995–2017 (Figure 3). Since we had no information about the associated risk behavior of the respective individuals, we could not associate cluster formation with risk behavior. However, geographic location was available for analysis but was not associated with cluster formation.

### Evolutionary Rates and Dates of HIV-1 Subtype C in Ethiopia

To comprehensively describe the Ethiopian HIV-1 epidemic, we further performed analyses on the three large clusters (C-EA-259, C-EA-148, and C′-ET-153). Root-to-tip analysis indicated a temporal signal in the three data sets (correlation coefficient of 0.53, 0.43, 0.47 for C-EA-259, C-EA-148, and C′-ET-153, respectively).

First, we estimated the tMRCA of the transmission clusters and the C-EA clade. The clade tMRCA represents the date of the origin of the circulating subtype C clade in the region. In contrast, the estimated tMRCA of the Ethiopian transmission clusters should approximate the introductions and local spread of the viral strains in the country (Dalai et al., 2009; Esbjornsson et al., 2011). Based on the inferred tMRCA, the posterior median estimates of C-EA-259 (1975, 95% HPD: 1970–1979) and C-EA-148 (1976, 95% HPD: 1963–1985) were older than the estimate for the C′-ET transmission cluster (1983, 95% HPD: 1975–1988; Table 1). The median root tMRCAs for C-EA clade was estimated to be 1971 (95% HPD: 1966–1976).

**Table 1:** Population dynamics and evolutionary estimates for subtype C cluster in Ethiopia.

	Subtype C clade/Cluster		
	C-EA-259	C-EA-148	C′-ET-153
Sequences from Ethiopia (n)	213	124	124
Range of collection (year)	1988–2017	1996–2017	1995–2017
Mean coefficient of variation	0.24	0.23	0.33
Median evolutionary substitution rate (95% HPD)^1^	1.76 (1.49–2.00)	1.74 (1.11–2.40)	1.83 (1.33–2.32)
Median year of tMRCA (95% HPD)	1975 (1970–1979)	1976 (1963–1985)	1983 (1975–1988)
Median rate of population growth (95% HPD)^2^	0.66 (0.51–0.81)	0.61 (0.38–0.86)	0.80 (0.53–1.10)
Median epidemic doubling time (years) (95% HPD)^3^	1.05 (0.86–1.36)	1.12 (0.81–1.82)	0.86 (0.63–1.31)
Maximum effective reproductive number (R_e_)^4^	6.13 (95% HPD, 3.53–10.14)	3.93 (95% HPD, 1.88–7.07)	4.88 (95% HPD, 2.57–8.57)
Basic reproductive number (R_0_)^5^	4.30 (95% HPD: 3.55–4.05)	4.05 (95% HPD: 2.90–5.25)	5.00 (95% HPD: 3.65–6.50)
Median become uninfectious rate (95% HPD)^6^	0.13 (0.06, 0.20)	0.19 (0.08, 0.29)	0.21 (0.09, 0.33)

1 Median number of substitutions/site/year × 10^−3^.

2 Median population growth rate (*r*) per year, determined in BEAST v1.10.4 using a logistic tree prior.

3 The time (years) required to double the effective number of infections (*λ*), calculated as *λ* = ln(2)/*r*, where *r* is the population growth rate.

4 R_e_ (effective reproductive number) which reflect the average number of secondary infections from an infected individual at any given time during the epidemic.

5 Basic reproductive number (R_0_) which reflects the average number of infections generated by an infected individual in a population where all individuals are susceptible to infection calculated by using the formula R_0_ = *rD* + 1(56) (where *r* is the population growth rate and *D* is the average duration of infectiousness period).

6 Become uninfectious rate, which reflects the inverse of the time duration of being infectious, in the unit of years.

The median estimated evolutionary rate was in the range 1.76–1.82 × 10^−3^ substitutions/site/year for the three clusters, with overlapping 95% HPD intervals (Table 1), indicating no significant difference of evolutionary rates among the three clusters.

### Temporal Dynamics of Viral Transmission

**Figure 4:**
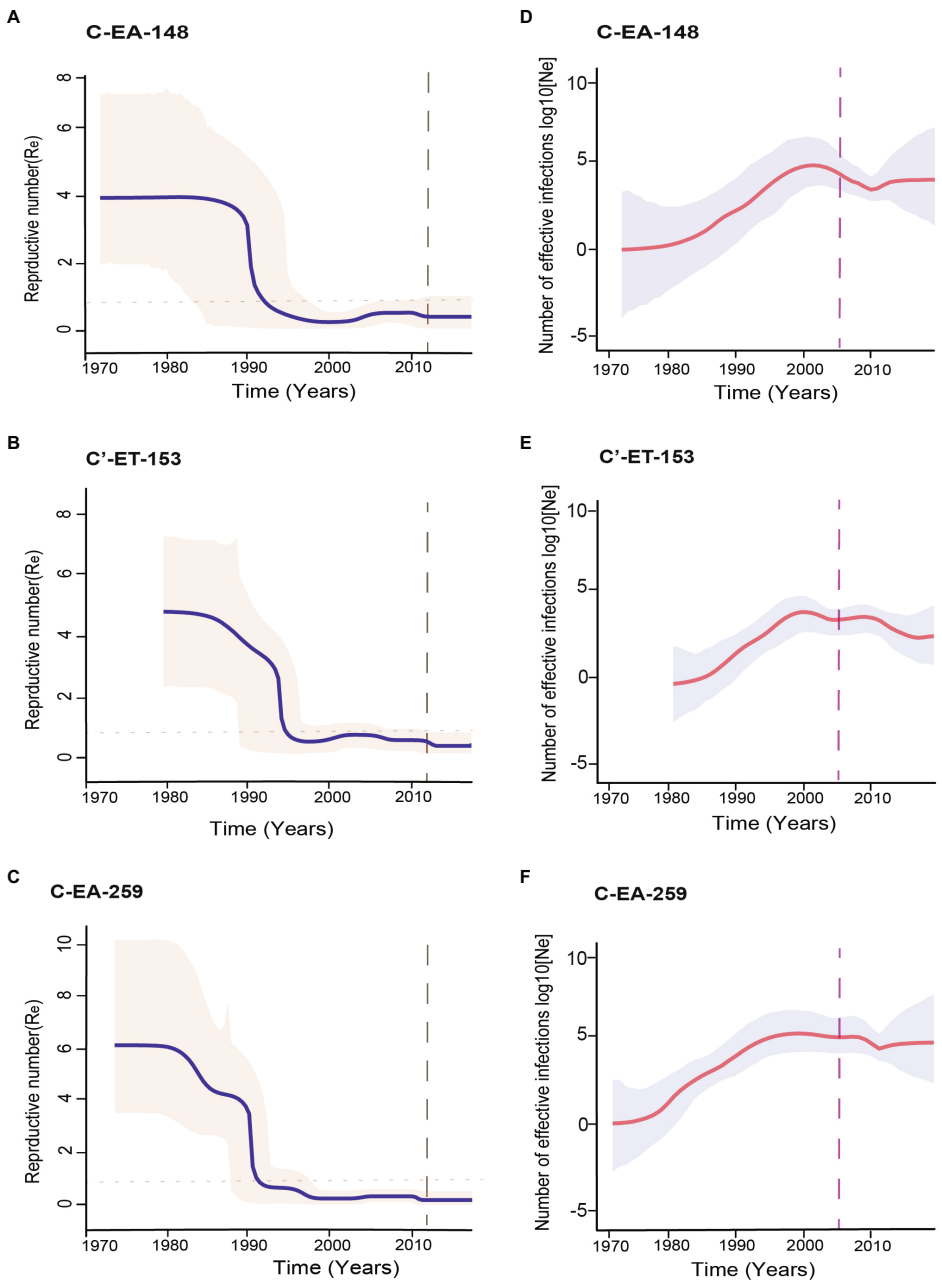
Population dynamics of the HIV-1 epidemic in Ethiopia using the three major clusters (C-EA-259, C-EA-148, and C’ET-153). **(A–C)** The temporal dynamics of effective reproductive number (R_e_) using the Bayesian birth–death model, the median R_e_ are shown by the continuous blue line, and indicated in a pink shade is the 95% highest probability density (HPD) intervals. The gray dashed line indicates the last coalescent event reported by the lineage through time (LTT) analysis. The horizontally dotted line represents the epidemiological threshold (Re = 1). **(D–F)** The median estimates of the effective population size (N_e_) over time using the Bayesian skygrid model. The red line shows the median logarithmic effective population size (N_e_) over viral generation time (*t*), representing effective transmissions, and the gray shade indicates the 95% highest probability density (HPD) intervals. The pink dashed line represents the time of antiretroviral therapy (ART) introduction in Ethiopia.

Direct estimation of the temporal dynamics of the effective reproductive number, R_e_, was performed using the BDSKY model. The BDSKY analysis assumed a piecewise constant R_e_, changing over six equidistance intervals between the tMRCA and the most recent sampling. The R_e_ showed similar dynamics for the three clusters. From the start until the beginning of the 1990s, R_e_ remained consistently high (R_e_ > 1) and dropped below the epidemiological threshold (R_e_ < 1) at the mid-1990s and remained below one till recent years (Figures 4A–C). In all three clusters, we observed the maximum R_e_ values (4-6) during the early period (before 1990) of the epidemic (Table 1).

The posterior median estimates of the become non-infectious rate obtained for each cluster were of 0.13 (95% HPD: 0.06–0.20) for C-EA-259, 0.19 (95% HPD: 0.08–0.29) for C-EA-148, and 0.21 (95% HPD: 0.09–0.33) for C′-ET-153, which translates to an infectious period of ~8 years for C-EA-259 and ~5 years for the other two clusters. Despite the longer infectious period estimated for C-EA-259, the overlapping HPD indicates no significant differences among clusters.

We performed different sensitivity analyses to explore the robustness of our BDSKY estimates. We used different values for the mean of the become non-infectious rate prior (δ) going from 6 months to 10 years (*δ*: 2, 1, 0.5, 0.2, 0.125, and 0.1). We also performed the sensitivity analysis by estimating the effective reproductive number for six and ten equally spaced intervals between tMRCA and the most recent sample. We obtained similar results for all analyses. R_e_ was consistently >1 for the early period until the early 1990s, followed by a decline to R_e_ < 1 after the mid-1990s.

We further performed phylodynamic analysis using the Bayesian Skygrid model to estimate the temporal characteristics of the HIV-1 epidemic in Ethiopia. We analyzed the three Ethiopian clusters and estimated the change in the effective population size (N_e_) through time, representing the change in the total number of infections contributing to new cases. The Bayesian skygrid inference revealed a rapid increase in N_e_ for all the three clusters from the initial introduction period until shortly before the year 2000, followed by a decline and stabilization in N_e_ until recent years (Figures 4D–F). We also determined the population growth rate (*r*), the rate of increase in the effective population size with time, using the logistic growth model of the coalescent parametric model. The median growth rate was 0.66, 0.61, and 0.80 year^−1^ for clusters C-EA-259, C-EA-148, and C′-ET-153, respectively, with overlapping HPD intervals (Table 1).

We also estimated the mean coalescent-based basic reproductive number (R_0_) values for each cluster from the logistic growth model using the formula R_0_ = rD + 1 (Pybus et al., 2001; where r is the population growth rate and D is the average duration of infectiousness period). Assuming an average infectious period of 5 years, R_0_ was in the range 4.0–5.0 for the three clusters, all with overlapping 95% HPD intervals (Table 1).

## Discussion

In this study, we analyzed a large dataset of HIV-1 *pol* sequences collected from different regions of Ethiopia during more than 30 years. We used both Bayesian coalescent and birth–death models to characterize the dynamics of the HIV-1 epidemic in the country. Overall, our analysis confirms that strains of two subtype C clades are circulating in Ethiopia, supporting the hypothesis that the HIV-1 epidemic in Ethiopia is the result of at least two independent HIV-1 introductions from eastern and southern African countries (Delatorre and Bello, 2012). Moreover, the phylodynamic analyses revealed that the epidemic dynamics in Ethiopia were characterized by an expanding epidemic growth from the start of the epidemic until the mid-1990s, followed by a sharp decline in HIV-1 transmissions. The decline in R_e_ occurred many years before introducing ART and coincided with early behavioral, preventive interventions, and public health awareness campaigns implemented in Ethiopia.

R_e_ is a proxy for HIV incidence and describes the transmission dynamics; R_e_ > 1 means that the epidemic is growing, R_e_ < 1 shows the epidemic is declining, while R_e_ = 1 shows that the epidemic is stabilizing (Stadler et al., 2013). Our phylodynamic analysis showed that the three clusters followed similar epidemic trends. The BDSKY model indicated epidemic growth (R_e_ > 1) from the 1970s to the early 1990s. The basic reproductive number (R_0_) and mean initial R_e_ were comparably high for the clusters, indicating an early exponential epidemic growth. Similarly, a high epidemic growth rate was estimated for each cluster (0.61–0.80 year^−1^) and a steady increase in N_e_ until the beginning of 2000, highlighting the upward trend of HIV transmissions in Ethiopia during the period.

The exponential epidemic growth observed in our analyses is consistent with retrospective serological data, which showed a massive increase of HIV infections among risk populations in Addis Ababa and cities along the main trading routes in Ethiopia during this early period. An extensive survey on FSWs operating in the main trading routes of Ethiopia in 1988 reported an HIV-1 prevalence between 5.3% and 38.1% (Mehret et al., 1990c). Studies performed in the capital Addis Ababa, 1988–90, showed an increase in prevalence from 25% to 54%, and 13% to 18% among FSWs and LDTDs, respectively (Khodakevich et al., 1990; Mehret et al., 1990a,c; Kebede et al., 2000), and 12%–18% among soldiers 1990–1993. Similarly, an increase in HIV prevalence among pregnant women attending antenatal care clinics (ANC) in Addis Ababa (4.6%–10.5%, 1989–90; Kebede et al., 2000) indicated extensive spread in the population.

During the early years, the rapid epidemic increase was most likely due to lack of awareness of HIV, high mobility among FSWs, high-risk sexual behavior, high STI prevalence among the general population (Desta et al., 1990; Mehret et al., 1990b; Negassa et al., 1990), while no prevention interventions were in place. The increased population movement following considerable urbanization and political instability in the country during this early period might also have contributed to the high HIV prevalence and epidemic spread (Hladik et al., 2006; Esbjornsson et al., 2011).

Although there is a lack of data that can describe the HIV epidemic on a national scale, different studies have shown a decline in new infections since the mid-1990s, corroborating our results (Kebede et al., 2000; Tsegaye et al., 2002; Wolday et al., 2007). The decline in HIV prevalence among young adult women (15–24 years) represents a well-established indicator of epidemic decline. It measures the frequency of relatively recent infections and is less influenced by death (Tsegaye et al., 2002). The HIV prevalence trend among young women (15–24 years) attending ANCs in Addis Ababa between 1995 and 2003 declined significantly from 24.2% to 12.9% (Tsegaye et al., 2002; Wolday et al., 2007). Moreover, there was a sharp decline in HIV prevalence among young blood donors in Addis Ababa and nine other towns during this period (Kebede et al., 2000).

Due to a lack of comprehensive data, it has not been easy to obtain estimates of the national incidence trend in Ethiopia. However, a study done to assess the temporal trend among pregnant women who attended the ANCs in the capital Addis Ababa, assessing >7,000 serum specimens collected 1995–2003, showed a significant decline in the HIV-1 incidence rate (from 7.7% to 2.0%, 1995–2003; Wolday et al., 2007). The reduction was substantial among young ANC attendees (aged 15–19 years), indicating an epidemic decline (7.8% to 0.0%, 1995–2003; Wolday et al., 2007). A mathematical modeling study also demonstrated a substantial reduction in the HIV incidence in Ethiopia after 1995 with an estimated annual decline of 6.3% per year, resulting in a total decrease of 77% between 1990 and 2016 (Deribew et al., 2019).

The early decline in the HIV transmissions observed in our study and documented in serological surveys coincide with the change of sexual behavior, prevention, and better control of other sexually transmitted infections (STIs) achieved through the sustained public education and mobilization campaigns. Ethiopia was one of the first countries in sub-Saharan Africa to introduce a task force to prevent and control HIV/AIDS and STI infections, including a national plan for the HIV epidemic response intervention (Zewdie et al., 1990; Kebede et al., 2000; Kloos and Mariam, 2000; Okubagzhi and Singh, 2002). During the early 1990s, Ethiopia had implemented a wide range of HIV prevention and information programs. Implementation of several behavioral interventions and awareness programs took place using the national media, schools, and public gatherings (Hadgu et al., 1990; Zewdie et al., 1990). These programs mainly focused on sustained health education, risk reduction, condom promotion, and prevention and control of STIs (Zewdie et al., 1990; Okubagzhi and Singh, 2002).

The national survey data on behavioral risk factors in Ethiopia are limited. However, different program reviews (1989–1991) and two nationwide surveys on condom use (1987–1993) revealed that these interventions led to changes in sexual risk behavior and increased knowledge about HIV/AIDS. Moreover, the intervention increased condom use and substantially reduced both non-regular partner and STI (Mehret et al., 1996). Similarly, another study showed condom use increased, and non-regular partners decreased among high school students in Addis Ababa and Gondar in the period after 1990 (Kebede et al., 2000). Moreover, a study among male factory workers in Ethiopia showed a change in sexual risk behavior (Mekonnen et al., 2003). Although it is difficult to quantify the impact of the different interventions on HIV incidence, it is reasonable to assume that the various prevention programs impacted HIV transmissions.

Several other studies outside Ethiopia have reported a significant decline in HIV prevalence after behavioral interventions (Martin, 1987; Hessol et al., 1989; Nelson et al., 1996; Stoneburner and Low-Beer, 2004; Halperin et al., 2011). A study in Uganda and Zimbabwe showed a significant decline in HIV prevalence after 1990, resulting from public health intervention on reduced sexual risk behavior (Stoneburner and Low-Beer, 2004; Halperin et al., 2011). Similarly, behavioral interventions resulted in a substantial decrease in HIV transmissions among MSM in Europe and North America in the mid-1980s and heterosexuals in Thailand in the early 1990s (Martin, 1987; Hessol et al., 1989; Nelson et al., 1996; Hué et al., 2005). In line with our results, a comprehensive review of empirical and modeled HIV incidence trends across 20 countries in Sub-Saharan Africa, 1990–2012, revealed a decline in incidence commenced before introducing ART programs, highlighting the significance of behavioral intervention in reducing HIV transmissions (Taaffe et al., 2014).

**Figure 5:**
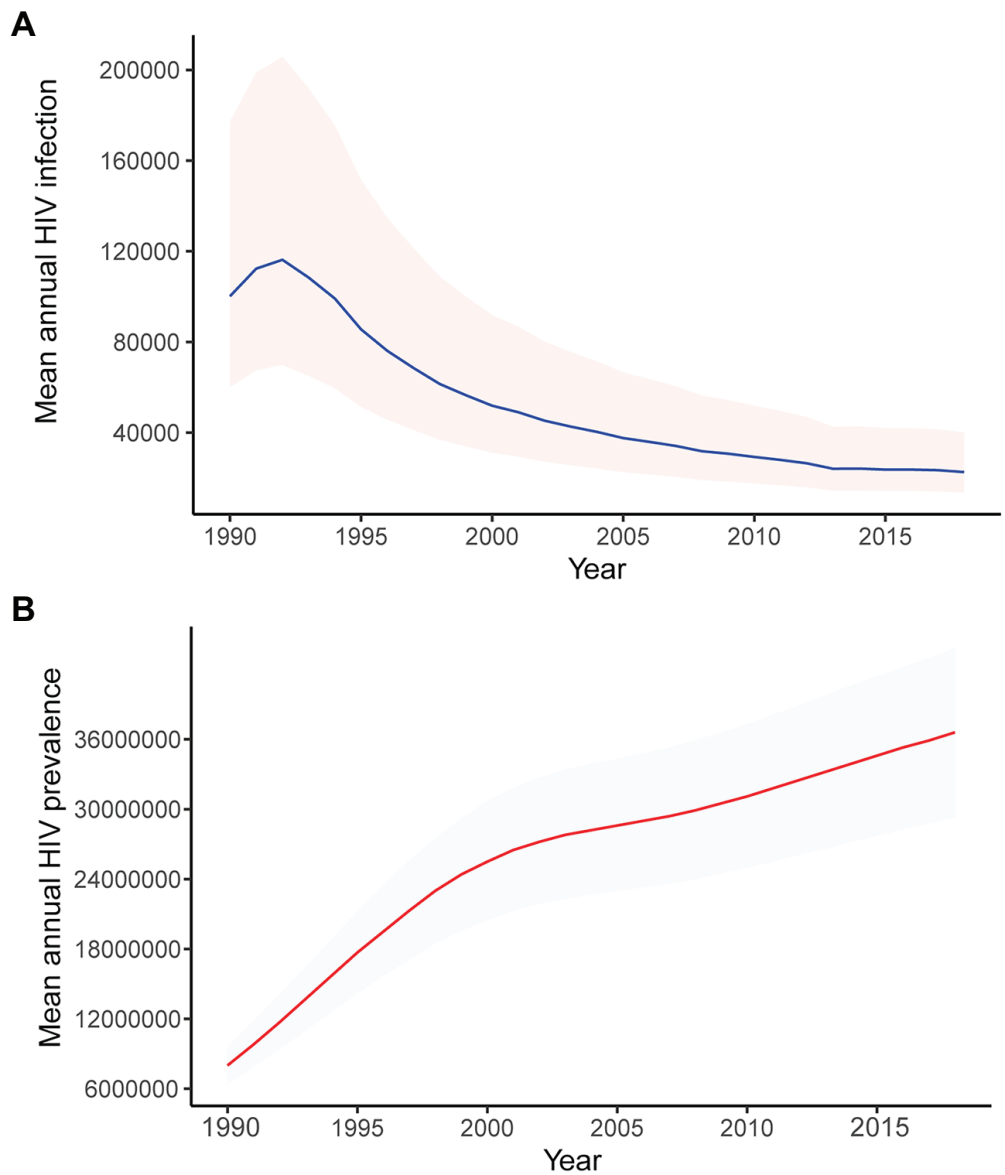
Modeled mean annual HIV **(A)** incidence and **(B)** prevalence, 1990–2018. Data obtained from UNAIDS.

The trends of the phylodynamic analyses (Figure 4) are in concordance with the UNAIDS HIV incidence and prevalence modeled estimates (Figure 5),^4^ showing a high incidence and prevalence during the years before 1990–1995, followed by a decline in incidence and stabilization in prevalence.

Thus, our results align well with published serological and epidemiological trends in Ethiopia. The epidemic decline coincides with the timing of behavioral interventions in Ethiopia, suggesting a link between the early decline of HIV spread and behavioral interventions many years before the implementation of ART in the country. However, the introduction of ART, which has proved to successfully suppress HIV replication and reduce the risk of onward transmissions, has significantly contributed to reducing HIV transmission, mortality, and maintaining the epidemic decline (Cohen et al., 2011).

The phylogenetic analysis confirms that strains of two HIV-1 subtype C clades (C′-ET and C-EA) are circulating in Ethiopia, suggesting that the HIV epidemic in Ethiopia arose by at least two independent introductions of founder strains from the eastern and southern African countries, respectively (Pollakis et al., 2003; Delatorre and Bello, 2012). Previous studies have defined several distinct subtype C clades that, in most cases, are associated with geographical regions (Thomson and Fernandez-Garcia, 2011). In the case of the Ethiopian lineages, they represent a southern African clade (where the Ethiopian C-SA sub-clade named C′-ET is more or less confined to Ethiopia) and an eastern African clade (C-EA). Our findings align with previous studies showing a distinct phylogeographic subdivision of the HIV-1 subtype C circulating in east, central, and southern African countries (Abebe et al., 2000; Pollakis et al., 2003; Thomson and Fernandez-Garcia, 2011; Delatorre and Bello, 2012).

The transmission cluster analysis indicated that the Ethiopian sequences formed large clusters, indicative of a few major introductions or expansions in the country. The three Ethiopian clusters described here were mixed regarding collection sites, suggesting intermixing of the HIV epidemic in Ethiopia. Different socio-cultural and behavioral factors might also contribute to cluster formation, and assessing these factors are essential for designing HIV-1 transmission preventive strategies. However, the sequences obtained from the public database do not contain information on risk factors, sociodemographic, and other clinical information. Hence, further analysis on factors associated with cluster formation was not possible to discern in this study.

Sequences of Burundi dominated the basal root of the large monophyletic C-EA clade incorporating more than 90% of the Ethiopian sequences, which is in line with a previous study showing that the C-EA clade likely had its origin in Burundi (Delatorre and Bello, 2012). Moreover, the basal root of the monophyletic clade defining the C′-ET clade was dominated by sequences from southern African countries, possibly reflecting the origin of this clade from southern African countries. However, our analysis could not identify the exact countries. Interconnectivity between populations due to geographic proximity has been an essential factor for the spread of HIV across African countries (Wilkinson et al., 2015, 2016; Faria et al., 2019). However, the large distances and cultural interconnectivity between Ethiopia, Burundi, and southern African countries suggest that other factors were in play. Population movements (due to unknown reasons) could have played a role in the introduction of HIV-1 subtype C to Ethiopia, similar to those observed in other parts of Africa (Gray et al., 2009).

Estimating the date of origin and timing of transmissions of HIV is essential to understanding the dynamics of HIV spread. Here, integral to our analysis of HIV transmission dynamics, we also obtained the tMRCA of the C-EA and C′-ET clades in Ethiopia. The molecular dating analysis suggested that the introduction of the C-EA clade took place more than a decade before the first reported AIDS case in Ethiopia. The dating is plausible considering that AIDS symptoms typically arise 6–10 years after infection. Moreover, our tMRCA estimates coincide with estimates of the introductions of the C-EA clade in other Eastern Africa countries, including Kenya, Tanzania, and Uganda (Delatorre and Bello, 2012), and are consistent with previous estimates for subtype C introduction in Ethiopia (Delatorre and Bello, 2012; Mir et al., 2018). Notably, this period also coincided with a large population migration from Burundi, which could have played a crucial role in disseminating the C-EA clade to Eastern Africa countries (Delatorre and Bello, 2012).

In contrast, the tMRCA of C′-ET was estimated at the beginning of the 1980s and is likely the result of a single introduction. This period coincided with the years of socio-political changes in the southern African countries and is associated with a steep growth of the HIV epidemic and viral migrations within southern African countries (Wilkinson et al., 2015, 2016).

To our knowledge, this study represents the most comprehensive study concerning the HIV epidemic in Ethiopia to date. It employs a large number of HIV-1 *pol* sequences collected during more than 30 years (1986–2017) from different geographical locations in Ethiopia. Moreover, we used state-of-art phylogenetic and phylodynamic methods to investigate the dynamics of the epidemic. Like many other molecular epidemiology studies, we incorporated HIV-1 *pol* gene sequences deposited in public databases in our analysis. As new HIV infections are recorded, more sequencing will allow to keep track of the ongoing transmission dynamics. The total sampling density was low, mainly due to a generally low sequencing coverage in Ethiopia, compared to the country’s total number of infected individuals. Thus, the transmission clusters identified here cannot fully represent Ethiopia’s entire HIV-1 transmission networks. Moreover, we based our analysis on HIV-1 *pol* sequences, representing the most sequenced HIV region due to the numerous published HIVDR studies. Although the HIV-1 *pol* fragment has sufficient phylogenetic signal for phylogenetic analysis of HIV (Hué et al., 2004), longer sequences, including whole genome sequences, may have provided a more informative inference of the HIV-1 molecular epidemiology and transmission history. Finally, the sequences used in this analysis lacked associated information, such as clinical, demographic, risk population assignment, or socio-economic data and, hence, we could not perform a detailed analysis of associated risk factors for HIV transmissions in our study.

In summary, we have employed state-of-art phylogenetic and phylodynamic approaches to describe the molecular epidemiology of HIV in Ethiopia. Our findings indicate that two distinct HIV subtype C strains were introduced in Ethiopia at the beginning of the 1970s and 1980s, followed by rapid epidemic growth until it started to decline in the mid-1990s, a decade before ART roll-out in Ethiopia. The sharp decline coincided with several behavioral prevention interventions and awareness campaigns. Our finding highlights the significance of scaling up behavioral and risk reduction interventions in addition to ART scale-up in the HIV/AIDS control strategy.

## Data Availability Statement

The datasets presented in this study can be found in online repositories. The names of the repository/repositories and accession number(s) can be found in the article/Supplementary Material.

## Author Contributions

DA, PB, and PM conceived and designed the study. DA, YK, PM, and TB coordinated the laboratory tests. DA, LE-G, SS, DK, and PM conducted the phylogenetic and phylodynamic analysis and interpreted the results. DA and PM wrote the manuscript. All authors reviewed the draft and contributed important intellectual content to the final version. All authors agreed and approved to the published version of the manuscript.

## Author Disclaimer

The findings and conclusions in this paper are those of the authors and do not necessarily represent the official position of the funding agencies.

## Funding

PM was supported by the Swedish Research Council (grant numbers 2019-05235 and 2020-02344), Österlund Foundation, and a donation to PM and PB through the Medical Faculty, Lund University, supported the research. LRE-G and DK were supported by the Max Planck Society. The computations were enabled by resources in project SNIC 2021-5-69 provided by the Swedish National Infrastructure for Computing (SNIC) at UPPMAX, partially funded by the Swedish Research Council through grant agreement no. 2018-05973.

## Conflict of Interest

The authors declare that the research was conducted in the absence of any commercial or financial relationships that could be construed as a potential conflict of interest.

## Publisher’s Note

All claims expressed in this article are solely those of the authors and do not necessarily represent those of their affiliated organizations, or those of the publisher, the editors and the reviewers. Any product that may be evaluated in this article, or claim that may be made by its manufacturer, is not guaranteed or endorsed by the publisher.

## Footnotes

^1^ http://www.hiv.lanl.gov

^2^ http://jphmm.gobics.de/submission_hiv

^3^ https://github.com/laduplessis/bdskytools

^4^ https://aidsinfo.unaids.org

## Supplementary Material

Supplementary Table S1 The GenBank accession numbers and the year of sampling of sequences used for this study.
